# Association of temporal discounting with transdiagnostic symptom dimensions

**DOI:** 10.1038/s44184-024-00060-3

**Published:** 2024-04-16

**Authors:** Kristof Keidel, Xiaping Lu, Shinsuke Suzuki, Carsten Murawski, Ulrich Ettinger

**Affiliations:** 1 Department of Psychology, University of Bonn, Bonn, Germany; 2 Centre for Brain, Mind and Markets, Department of Finance, The University of Melbourne, Carlton, Vic Australia; 3 Faculty of Social Data Science, Hitotsubashi University, Tokyo, Japan; 4 HIAS Brain Research Center, Hitotsubashi University, Tokyo, Japan

**Keywords:** Human behaviour, Cognitive neuroscience, Psychiatric disorders

## Abstract

Temporal discounting (TD), the tendency to devalue future rewards as a function of delay until receipt, is aberrant in many mental disorders. Identifying symptom patterns and transdiagnostic dimensions associated with TD could elucidate mechanisms responsible for clinically impaired decision-making and facilitate identifying intervention targets. Here, we tested in a general population sample (*N* = 731) the extent to which TD was related to different symptom patterns and whether effects of time framing (dates/delay units) and monetary magnitude (large/small) had particularly strong effects in people scoring higher on specific symptom patterns. Analyses revealed that TD was related to symptom patterns loading on anxious-depression and inattention-impulsivity-overactivity dimensions. Moreover, TD was lower in the date than the delay version and with higher magnitudes, especially in people scoring higher on the inattention-impulsivity-overactivity dimension. Overall, this study provides evidence for TD as a transdiagnostic process across affective and impulsivity-related dimensions. Future studies should test framing interventions in clinical populations characterized by impulsivity.

Preregistration: This research was preregistered at https://osf.io/fg9sc.

## Introduction

When faced with a decision between a smaller-sooner reward and a larger-later reward (*intertemporal choice*), people devalue the future reward relative to the sooner reward as a function of delay until receipt, a phenomenon known as *temporal discounting* (TD)^1^. Steeper TD has been found in numerous mental disorders, including behavioral addictions^2–4^, substance use disorders^2,5,6^, major depression^7^, attention-deficit/hyperactivity disorder (ADHD)^8^ and bulimia nervosa^7^, while shallower TD occurs in anorexia nervosa^7^. Therefore, TD has been proposed as a transdiagnostic marker of mental disorders^7,9–11^.

Despite convincing evidence of aberrant TD in clinically diagnosed groups compared to healthy controls, few studies have examined transdiagnostic determinants, i.e., specific symptom clusters responsible for these relationships^7^. This is likely due to problems inherent in studying diagnostic categories. Specifically, in case-control studies, sample sizes are often limited, diagnostic groups are heterogenous, and controlling for treatment confounds is difficult^12,13^.

Dimensional approaches like the Research Domain Criteria framework (RDoC), which aims to identify common behavioral and biological dimensions of mental disorders^14–16^, or the Hierarchical Taxonomy of Psychopathology (HiTOP), which systematically describes and organizes psychopathological dimensions at different hierarchical levels^17,18^, may help overcome these issues. These build upon the observation that many symptoms are not specific but characterize a range of mental disorders. For instance, impulsivity has been robustly related to various internalizing and externalizing psychopathologies^19^. Also, anxiety symptoms play a role not only in anxiety disorders but also in other (internalizing) disorders like depression or obsessive-compulsive disorder (OCD)^20–22^. Additionally, it has been observed that many symptom patterns are dimensional in extending from clinical groups into the non-clinical population^23,24^. Therefore, to account for both overlaps across clinical disorders and varying symptom expressions in the population, fully-dimensional transdiagnostic approaches identify transdiagnostic dimensions by recruiting large unselected samples experiencing psychopathology to a varying extent and reducing dimensionality using factor analysis of self-report questionnaire responses^12,25,26^.

In the context of intertemporal choice, such an approach may help elucidate which transdiagnostic symptom patterns are associated with steeper or shallower TD efficiently and with limited psychopathology-related confounds. As a consequence, this may help explain inconsistent findings regarding associations of TD with disorders such as autism-spectrum disorders^27–30^ or schizophrenia^31–36^, which could be due to highly heterogenous symptom clusters^9^ or medication confounds^13^. Furthermore, a dimensional approach may help find interventions targeting aberrant TD by evaluating the effectivity of interventions depending on specific symptom pattern severity^9^. Specifically, previous studies have shown that TD can be influenced by experimentally manipulating task framing, incidental affective states, or prospection-related processes^37^. Some of these manipulations, e.g., presenting time until receipt of rewards in dates instead of delay units (date/delay effect)^38–40^, reduce TD not only in the general population but especially in individuals initially showing particularly steep TD, such as problematic alcohol users^41^ or persons with high positive schizotypy scores^42^. Therefore, finding transdiagnostic symptom dimensions that are associated with TD and that are particularly responsive to such manipulations could be of high therapeutic value.

To our knowledge, only one study has examined TD using a transdiagnostic dimensional approach, finding elevated TD in substance use, ADHD, depression, and anxiety, supporting the transdiagnostic nature of TD^11^. Here, we used a similar approach but administered a largely different set of questionnaires to test whether various psychiatric symptom patterns can account for individual differences in TD in the population. Importantly, since within-subject manipulations of TD might be an effective intervention method^9^, we manipulated time framing to investigate the date/delay effect and its association with psychiatric symptom patterns and transdiagnostic dimensions.

Our preregistered hypotheses were as follows:

1. We hypothesized that individual differences in psychiatric symptom patterns would be related to individual differences in TD. Specifically, we expected that higher scores in depression, anxiety, OCD, schizotypy, autism, and ADHD measures would be associated with steeper TD. Additionally, we expected that uncontrolled eating and emotional eating would be positively and eating-related cognitive restraint negatively associated with TD. For impulsivity, we expected positive associations of positive urgency, negative urgency, and lack of premeditation with TD.
2. We hypothesized that TD would be shallower when time was presented as dates (e.g., on November 30, 2022) compared to delay units (e.g., in 19 days), replicating the date/delay effect.

Furthermore, we conducted a preregistered explorative analysis to determine whether the date/delay effect was stronger in participants with higher levels in the symptom patterns examined.

Finally, in a non-preregistered explorative analysis following a reviewer’s comment, we also considered associations between symptom patterns and (i) TD model fit and (ii) the extent of the magnitude effect, i.e., the reduction in TD with larger compared to smaller monetary rewards^43^.

## Method

The study was approved by the University of Melbourne Ethics Committee (ID: 21719). All participants provided written informed consent for study participation and re-use of anonymized data.

### Design

The study was cross-sectional, conducted online on Qualtrics (https://www.qualtrics.com/) between 18/08/2022 and 15/12/2022.

### Sample and exclusion criteria

Participants were recruited via Prolific (https://www.prolific.co/). Data were sampled from the U.S. general population, aged 18–65 years and fluent in English. Participants were excluded from data analysis if they failed at least one of five attention checks in the questionnaires (further details: Supplementary Methods; preregistration: https://osf.io/fg9sc). They were paid a base sum of USD10 plus bonus (averaging USD3.05) depending on performance in a complex decision-making task reported elsewhere. Participation took 85 minutes on average.

### Intertemporal choice task

The Monetary Choice Questionnaire (MCQ)^44^ comprises 27 decisions between hypothetical smaller-immediate and larger-later rewards, which can be divided into three magnitudes based on the amounts of the larger-later rewards: small ($25 to $35), medium ($50 to $60), and large ($75 to $85). Participants completed two versions of the MCQ: a delay version where times were presented in days (e.g., “Would you prefer $14 in 0 days, or $25 in 19 days?”) and a date version where they were presented in dates (e.g., “Would you prefer $14 on November 11, 2022, or $25 on November 30, 2022?”). Decisions were presented one-by-one. Order of versions and items within versions were randomized.

### Cognitive ability task

We used the 16-item sample test of the International Cognitive Ability Resource (ICAR)^45^ to assess cognitive ability. It consists of four items each of the types verbal reasoning, letter and number series, matrix reasoning, and three-dimensional rotation.

### Self-report psychometric questionnaires

Participants completed a range of questionnaires assessing depression/anxiety/stress, trait anxiety, obsessive-compulsive tendencies, schizotypy, different types of disordered eating behavior (uncontrolled eating, cognitive restraint, emotional eating), autistic tendencies, ADHD symptoms, and impulsivity (negative urgency, lack of premeditation, lack of perseverance, sensation seeking, positive urgency). The questionnaires used are listed in the Supplementary Methods. Five nonsensical items were embedded in the questionnaires as attention checks (Supplementary Methods).

### Statistical analyses

G*Power 3.1^46^ was used for power analysis, all other analyses were conducted in R (version 4.2.2) using RStudio (version 2023.06.1). For estimation of discount rates, we used RStan (version 2.26.1). Significance tests were conducted applying a two-tailed alpha-level of 0.05. In all analyses in which the discount rates of both the delay version and the date version were used to test the same research question (i.e., hypothesis 1) employing the same statistical approach, we computed *p*-values using Bonferroni-Holm correction (*p*_c_) to account for testing across two dependent variables.

#### A priori power analysis

Given that associations between individual difference variables and TD are low^47^, we a priori assumed a two-tailed alpha-level of 0.05, a power of 80%, and a correlation effect size of *r* = 0.10. This power analysis yielded a target sample size of 782, which we rounded to 800.

#### Estimation of discount rates

Log-transformed discount rates were estimated based on the 54 intertemporal choices using Bayesian hierarchical modelling (e.g.^48^). Specifically, we estimated group- and participant-level parameters based on hyperbolic, exponential, and quasi-exponential functions and compared model fits. We note that deviating from our preregistration and based on recent evidence^49^, we did not compute Bayes factors but used leave-one-out cross-validation (LOO-CV)^50^ based on participant-level estimates to test which function type provided the best fit. Results revealed that predictive accuracies of the hyperbolic (elpd [expected log pointwise predictive density] estimate = −8,996.0, *SE* = 148.6) and exponential (elpd estimate = −9,189.3, *SE* = 150.4) models were higher than of the quasi-hyperbolic model (elpd estimate = −16,774.3, *SE* = 282.1). Differences between the hyperbolic and exponential models were small (i.e., below two times the *SE*), with the hyperbolic discounting yielding slightly better accuracies. Therefore, and in line with previous studies^51,52^, we estimated discount rates for the current study assuming a hyperbolic function.

We estimated a single hierarchical model accounting for differences in the two versions, estimating group- and participant-level parameters as well as their posterior distributions and point estimates for each version. The date/delay effect was computed as the difference of point estimates between versions (delay minus date). To test whether TD model fit differed between versions, we additionally estimated separate models for the two versions and compared model fits using LOO-CV. Results revealed that differences between the delay (elpd estimate = −4,330.0, *SE* = 80.1) and date (elpd estimate = −4,438.2, *SE* = 85.5) versions (elpd difference: −108.2, *SE* = 100.6) were small and non-significant (i.e., below two times the *SE*).

Then, to obtain log-transformed discount rates for the three different magnitudes of the MCQ, we estimated another Bayesian hierarchical model assuming a hyperbolic function, this time accounting for the differences in the three magnitudes. Thus, we estimated group- and participant-level parameters as well as their posterior distributions and point estimates for each MCQ magnitude (small, medium, large). The magnitude effect was computed as difference between point estimates of the small and large magnitudes.

#### Summary measures of ICAR, questionnaires and transdiagnostic dimensions

**Fig. 1:**
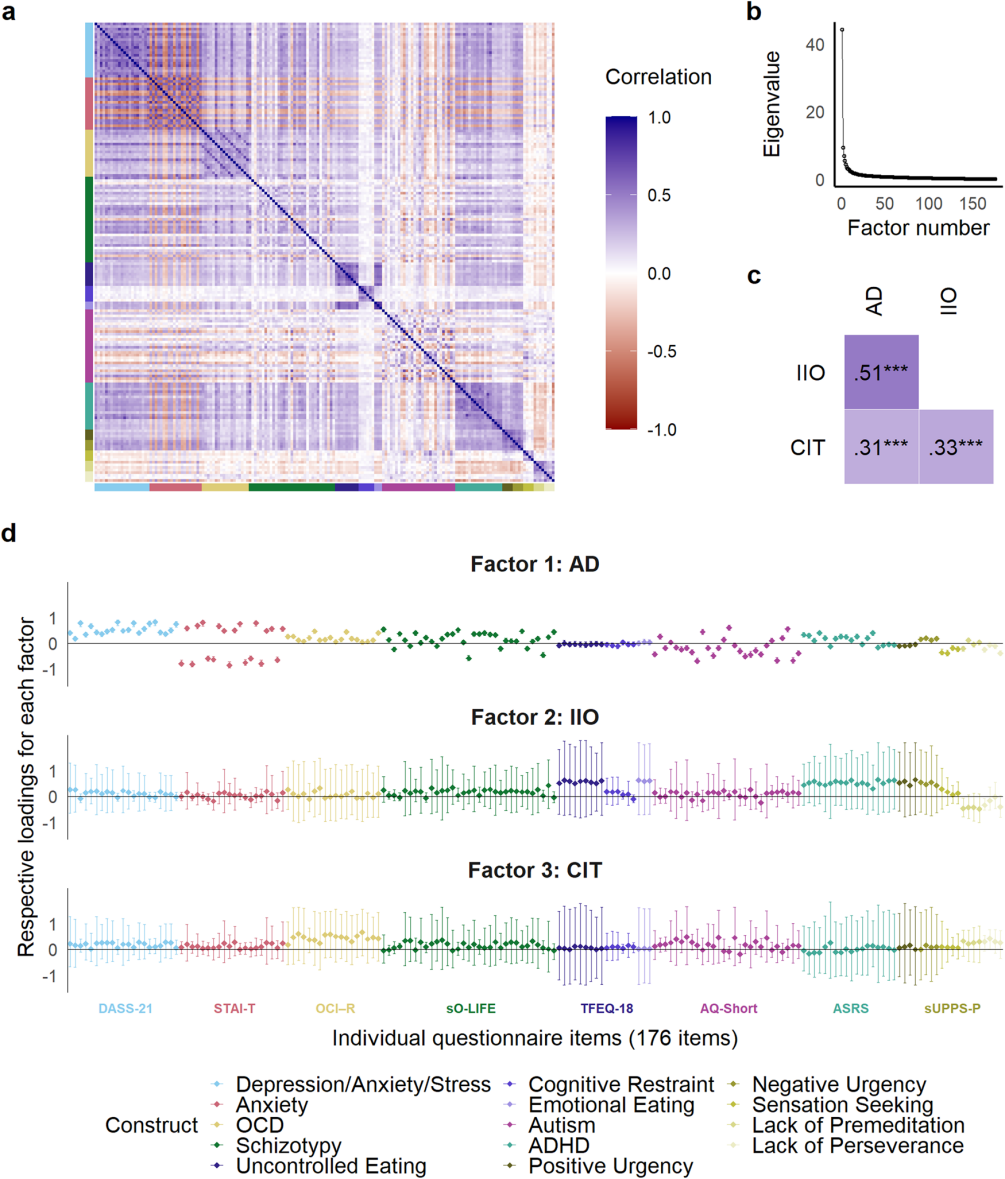
Results of exploratory factor analysis of clinical questionnaire items. **a** represents the heterogenous correlation matrix of the 176 items entered into the exploratory factor analysis; **b** represents the screeplot of the factor analysis; **c** represents Pearson correlations between participants’ factor scores; **d** represents the item loadings for each respective factor. Error bars represent 95% confidence intervals. ***p < 0.001. AD Anxious-Depression, IIO Inattention, Impulsivity and Overactivity, CIT Compulsive Behavior and Intrusive Thought, DASS-21 Depression Anxiety Stress Scales 21, STAI-T State-Trait Anxiety Inventory (trait version), OCI-R Obsessive-Compulsive Inventory–Revised, sO-LIFE Short Oxford-Liverpool Inventory of Feelings and Experiences, TFEQ-R18 Three-Factor Eating Questionnaire-R18, AQ-Short Autism-Spectrum Quotient Short, ASRS Adult ADHD Self-Report Scale, sUPPS-P Short UPPS-P Impulsive Behavior Scale.

We computed sum scores for the ICAR (sum of correct answers) and the questionnaires (sum of item scores). For the disordered eating and the impulsivity questionnaires, sum scores were computed for the subscales instead of the complete scales. To obtain transdiagnostic dimensions, we conducted a non-preregistered exploratory factor analysis with maximum-likelihood estimation^53–55^. We entered participants’ responses to the 176 questionnaire items into the factor analysis and, as item responses had different scale levels, computed a heterogenous correlation matrix (Fig. 1a). We determined the number of extracted factors using the Cattell-Nelson-Gorsuch (CNG) test^56,57^ based on the screeplot (Fig. 1b), applied an oblique factor rotation (oblimin), and extracted factor scores for each participant (Pearson correlations between factor scores in Fig. 1c). Confidence intervals of item loadings were estimated using bootstrapping with 1,000 iterations. Based on inspection of item loadings (Fig. 1d) and similar to previous studies^53–55^, we labelled the factors/dimensions “Anxious-Depression” (AD), “Inattention, Impulsivity and Overactivity” (IIO) and “Compulsive Behavior and Intrusive Thought” (CIT).

#### Psychiatric Symptom Patterns and TD

To examine associations between psychiatric symptom patterns and TD variables, we computed preregistered Pearson correlations. For these variables, outliers, i.e., values 3 *SD* above or below the mean were excluded from all analyses. To explore which predictor was strongest, we conducted preregistered linear regressions (ordinary least squares estimates) of discount rates on psychiatric symptom patterns. Due to the multicollinearity of depression/anxiety/stress and trait anxiety scores, we excluded trait anxiety scores from this analysis. Additionally, since Breusch-Pagan tests revealed at least some degree of heteroskedasticity in this and all following linear regression models, we calculated all models using heteroscedasticity-robust standard errors. Next, to examine associations between psychiatric symptom patterns and TD while controlling for covariates, we conducted non-preregistered linear regressions of discount rates in each version controlling for demographics and cognitive ability, i.e.: discount rates (delay/date) ∼ age + gender1 + gender2 + cognitive ability + scale. All regressors were *z*-standardized. Afterwards, we computed the same models using the transdiagnostic dimensions as predictors. Dimensions were entered in separate and joint models (i.e., controlling for the other dimensions).

#### Psychiatric symptom patterns and the date/delay effect

As preregistered, to test for the date/delay effect, we inspected with which probability the paired differences of discount rate posteriors of the two versions differed from zero using the Bayesian Estimation Supersedes the *t*-test (BEST; 30,000 MCMC iterations)^58,59^ and conducted a frequentist paired *t*-test. Then, to examine the association of psychiatric symptom patterns/dimensions with the date/delay effect, we conducted the same analyses as for the associations between psychiatric symptom patterns/dimensions and discount rates (Pearson correlations, linear regressions) but replaced discount rates with the date/delay effect.

#### Further explorative analyses: TD model fit and magnitude effect

In non-preregistered, explorative analyses, we analyzed whether the psychiatric symptom patterns/dimensions were associated with model fit. We used the individual point estimates of WAIC (widely applicable information criterion) as indicators of model fit and examined associations with psychiatric symptom patterns/dimensions using the same analyses as before (Pearson correlations, linear regressions).

Finally, we tested for the magnitude effect using a Bayesian approach (BEST; 30,000 MCMC iterations)^58,59^ to compare the differences between small vs. medium and medium vs. large log-transformed discount rates as well as a frequentist repeated-measures ANOVA with post-hoc pairwise comparisons (*t*-tests). To analyze associations between psychiatric symptom patterns/dimensions and the magnitude effect in TD, we used the magnitude effect as a variable of interest in the same analyses as before (Pearson correlations, linear regressions).

## Results

### Participants

Of 800 participants, *N* = 68 participants were excluded due to failed attention checks and *N* = 1 dataset was lost in Qualtrics, yielding a final sample size of *N* = 731.

360 (49.2%) participants reported a female, 351 (48.0%) a male and 20 (2.7%) diverse gender. 367 participants had a female (50.21%), 361 a male (49.38%) biological sex, and data of 3 participants (0.41%) were missing. Mean age was 37.6 years (*SD* = 11.4). Educational attainment was slightly higher than in the U.S. population (Supplementary Table 1). Self-reported ethnicity was distributed as follows: 61 Asian (8.34%), 51 Black (6.98%), 40 Mixed (5.47%), 534 White (73.05%), 25 other (3.42%), and data of 20 participants (2.74%) were missing.

### Psychiatric symptom patterns and TD

**Fig. 2:**
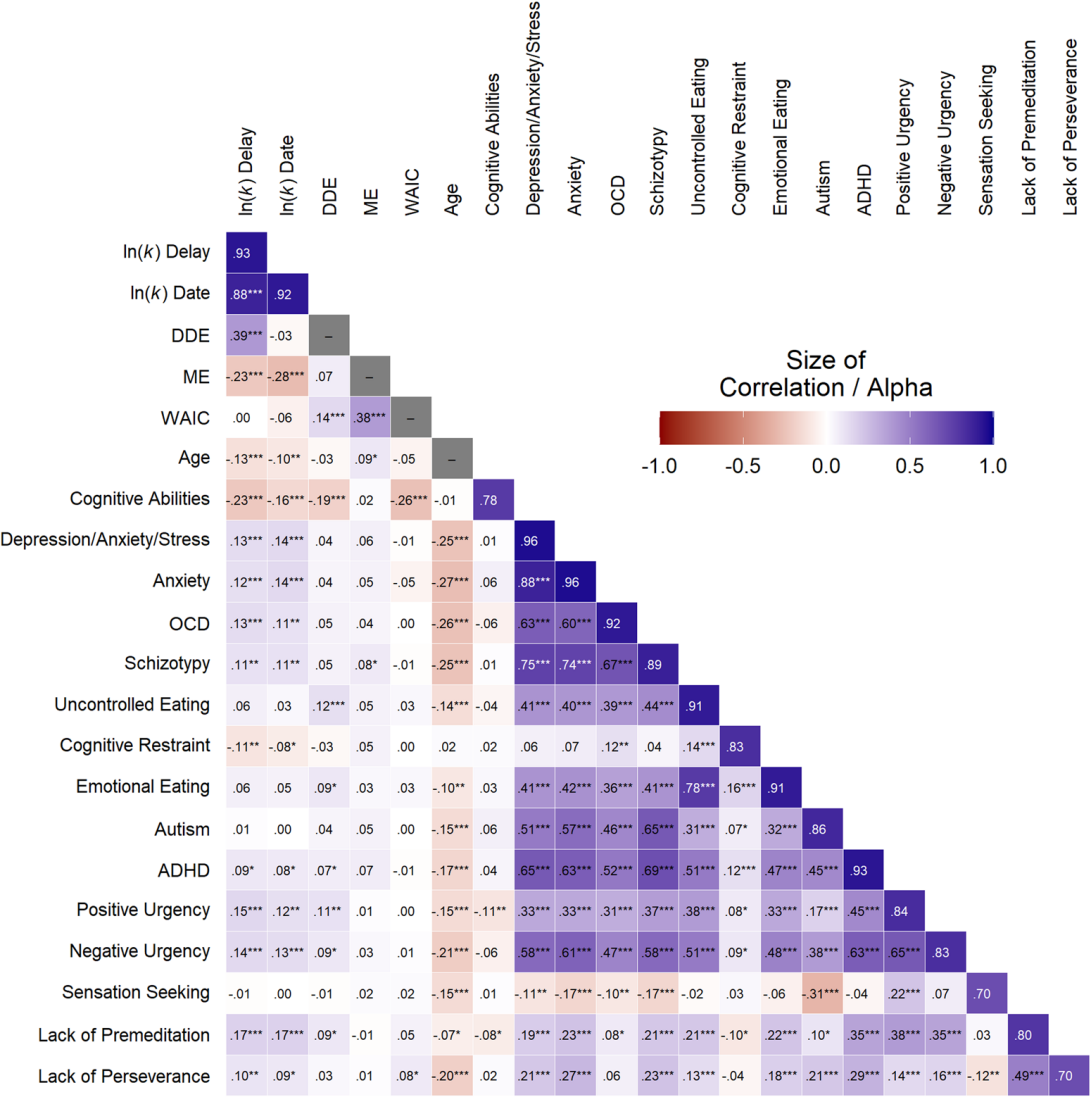
Pearson correlations between temporal discounting, demographic and questionnaire variables. ln(*k*) log-transformed discount rate, DDE date/delay effect, ME magnitude effect, WAIC widely applicable information criterion, OCD obsessive-compulsive disorder, ADHD attention-deficit/hyperactivity disorder. Values in the diagonal represent Cronbach’s alpha. *p < 0.05, **p < 0.01, ***p < 0.001 uncorrected.

Descriptive data of all summary measures are presented in Table 1. Pearson correlations (Fig. 2, Supplementary Table 2) revealed statistically significant positive associations of TD in either version with depression/anxiety/stress, trait anxiety, OCD, schizotypy, ADHD, and different impulsivity constructs (*r*s between 0.08 and 0.17, all *p*_c_ < 0.05), while only eating-related cognitive constraint was negatively related to TD (*r* = −0.11 and *r* = −0.08, both *p*_c_ < 0.05). Other correlations were not statistically significant. When controlling for the influence of all scales, effects of OCD (β = 0.13, 95% CI [0.02, 0.24], *p*_c_ = 0.046), eating-related cognitive restraint (β = −0.10, 95% CI [−0.18, −0.03], *p*_c_ = 0.02), and lack of premeditation (β = 0.12, 95% CI [0.03, 0.20], *p*_c_ = 0.01) were statistically significant in the delay and the effect of lack of premeditation (β = 0.14, 95% CI [0.05, 0.22], *p*_c_ = 0.006) was statistically significant in the date version (Supplementary Table 3).

**Table 1:** Descriptive values of main variables after exclusions

Variable	*N*	Range	*M*	*SD*	Sk
ln(*k*) Delay	731	−9.44 to −0.35	−4.77	1.80	−0.35
ln(*k*) Date	731	−9.35 to −0.60	−5.20	1.63	−0.21
Date/Delay Effect	718	−1.85 to 2.96	0.43	0.74	0.30
ln(*k*) Small	731	−8.89 to −0.64	−4.31	1.57	−0.63
ln(*k*) Medium	731	−9.01 to −0.72	−3.93	1.60	−0.29
ln(*k*) Large	731	−9.36 to −0.70	−5.29	1.71	−0.14
Magnitude Effect	726	−1.20 to 3.13	0.99	0.69	−0.08
Model Fit (WAIC)	720	3.39 to 50.69	21.47	8.85	0.73
Cognitive Abilities	731	0 to 16	8.29	3.54	0.15
Depression/Anxiety/Stress	729	21 to 79	38.38	13.79	0.60
Anxiety	731	20 to 80	44.92	14.26	0.15
OCD	726	18 to 67	31.94	11.56	0.91
Schizotypy	730	33 to 61	43.15	6.88	0.46
Uncontrolled Eating	731	9 to 36	17.59	6.21	0.75
Cognitive Restraint	731	6 to 24	12.57	4.05	0.33
Emotional Eating	731	3 to 12	5.88	2.75	0.61
Autism	730	35 to 101	66.43	11.91	−0.02
ADHD	729	18 to 85	43.31	13.69	0.26
Positive Urgency	723	4 to 14	6.32	2.47	0.93
Negative Urgency	731	4 to 16	8.08	3.17	0.37
Sensation Seeking	731	4 to 16	8.49	2.90	0.31
Lack of Premeditation	725	4 to 13	6.87	2.17	0.47
Lack of Perseverance	730	4 to 14	7.50	2.16	0.20

*N* represents the sample size after exclusions (values 3 *SD* above or below the mean). *ln(k)* log-transformed discount rate, *WAIC* widely applicable information criterion, *OCD* obsessive-compulsive disorder, *ADHD* attention-deficit/hyperactivity disorder, *Sk* skewness.

**Fig. 3:**
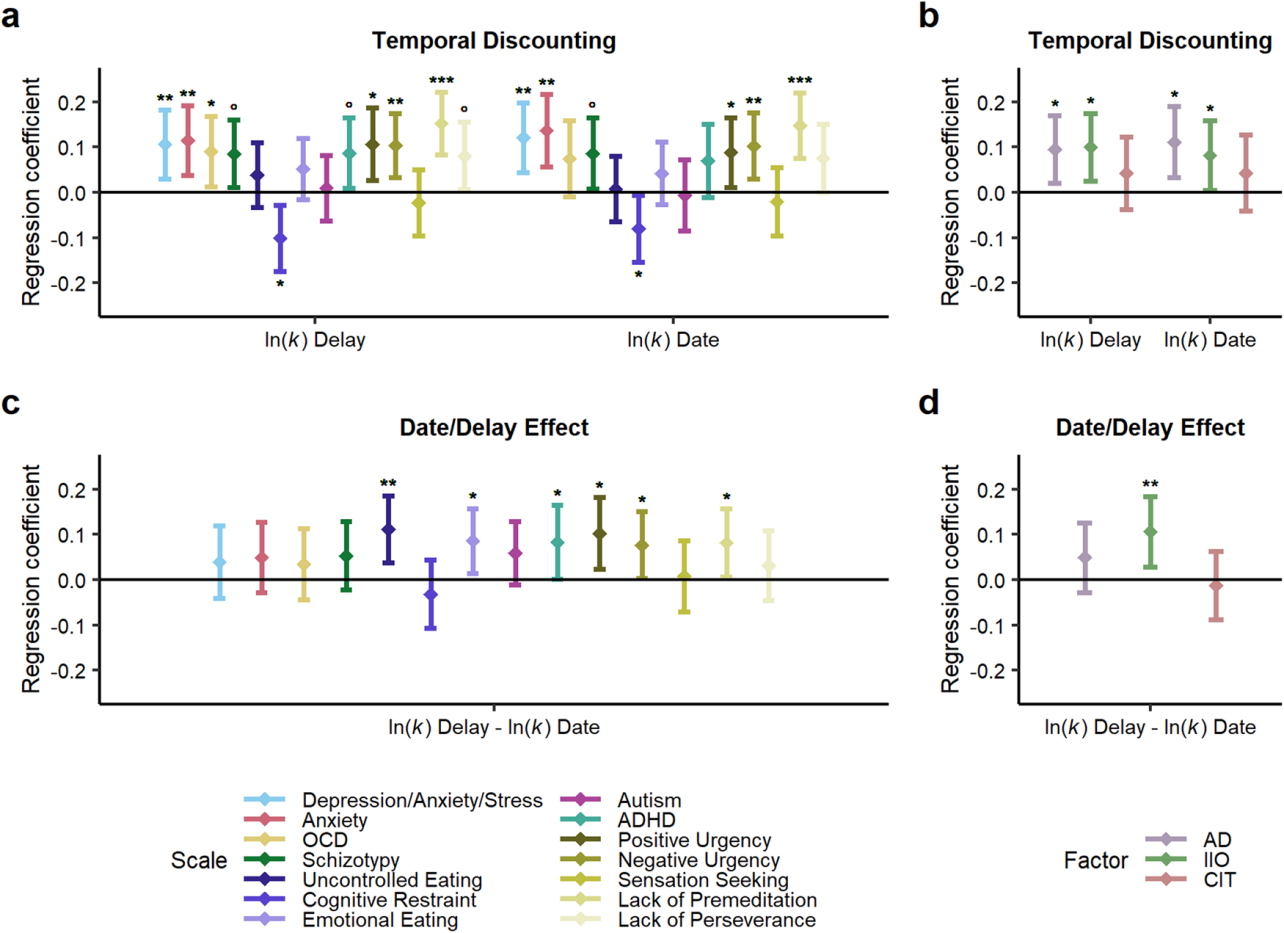
Associations between temporal discounting and psychiatric symptom patterns and the date/delay effect and psychiatric symptom patterns. Presented are separate regressions of discount rates in the two versions (**a**, **b**) or the date/delay effect (**c**, **d**) on different psychiatric symptom patterns (**a**, **c**) or transdiagnostic factors (**b**, **d**), controlling for gender, age and cognitive abilities. Error bars represent 95% confidence intervals. OCD obsessive-compulsive disorder, ADHD attention-deficit/hyperactivity, AD Anxious-Depression, IIO Inattention, Impulsivity and Overactivity, CIT Compulsive Behavior and Intrusive Thought *p < 0.05, **p < 0.01, ***p < 0.001, in **a** and **b** Bonferroni-Holm corrected for the two dependent variables tested; °*p* < 0.05, uncorrected.

In linear regressions controlling for gender, age, and cognitive ability (Fig. 3a, Supplementary Table 4), we found in both versions that depression/anxiety/stress (delay: β = 0.11, 95% CI [0.03, 0.18], *p*_c_ = 0.006; date: β = 0.12, 95% CI [0.04, 0.20], *p*_c_ = 0.004), trait anxiety (delay: β = 0.11, 95% CI [0.04, 0.19], *p*_c_ = 0.004; date: β = 0.14, 95% CI [0.06, 0.22], *p*_c_ = 0.002), positive urgency (delay: β = 0.11, 95% CI [0.03, 0.19], *p*_c_ = 0.02; date: β = 0.09, 95% CI [0.01, 0.17], *p*_c_ = 0.03), negative urgency (delay: β = 0.10, 95% CI [0.03, 0.17], *p*_c_ = 0.008; date: β = 0.10, 95% CI [0.03, 0.18], *p*_c_ = 0.008), and lack of premeditation (delay: β = 0.15, 95% CI [0.08, 0.22], *p*_c_ < 0.001; date: β = 0.15, 95% CI [0.08, 0.22], *p*_c_ < 0.001) were positively associated with TD, while eating-related cognitive restraint was negatively associated with TD (delay: β = −0.10, 95% CI [−0.17, −0.03], *p*_c_ = 0.01; date: β = −0.08, 95% CI [−0.15, −0.01], *p*_c_ = 0.04). Positive effects for OCD (β = 0.09, 95% CI [0.01, 0.17], *p*_c_ = 0.048) were statistically significant in the delay version only.

Next, using the transdiagnostic dimensions extracted from factor analysis (Fig. 3b), we found that, controlling for gender, age, and cognitive ability, AD (delay: β = 0.09, 95% CI [0.02, 0.17], *p*_c_ = 0.01; date: β = 0.11, 95% CI [0.03, 0.19], *p*_c_ = 0.01) and IIO (delay: β = 0.10, 95% CI [0.02, 0.17], *p*_c_ = 0.02; date: β = 0.08, 95% CI [0.00, 0.16], *p*_c_ = 0.04) were positively related to TD, while CIT (delay: β = 0.04, 95% CI [−0.04, 0.12], *p*_c_ = 0.60; date: β = 0.04, 95% CI [−0.04, 0.13], *p*_c_ = 0.60) was not. When additionally controlling for all dimensions, no effect was statistically significant (Supplementary Table 5).

### Psychiatric symptom patterns and the date/delay effect

The date/delay effect was replicated, as the paired difference (delay minus date) of discount rates had a probability of > 99.9% to be above zero (median paired difference = 0.39, 95% HDI [0.34, 0.45]), and the frequentist paired *t*-test was significant (*t*(730) = 13.66, *p* < 0.001, *g*_av_ = 0.25, 95% CI [0.22 to 0.29]).

A more pronounced date/delay effect was correlated with higher scores in uncontrolled eating, emotional eating, ADHD, positive urgency, negative urgency, and lack of premeditation (*r*s ranging between 0.07 and 0.12, all *p* < 0.05; Fig. 2, Supplementary Table 6). Using a single regression, uncontrolled eating (β = 0.14, 95% CI [0.01, 0.26], *p* = 0.03) and positive urgency (β = 0.12, 95% CI [0.00, 0.23], *p* = 0.048) persisted as statistically significant predictors (Supplementary Table 7). Associations between the date/delay effect and psychiatric symptom patterns were robust when controlling for gender, age, and cognitive ability (Fig. 3c, Supplementary Table 8), i.e., we found associations for uncontrolled eating (β = 0.11, 95% CI [0.04, 0.19], *p* = 0.003), emotional eating (β = 0.09, 95% CI [0.01, 0.16], *p* = 0.02), ADHD (β = 0.08, 95% CI [0.00, 0.16], *p* = 0.046), positive urgency (β = 0.10, 95% CI [0.02, 0.18], *p* = 0.01), negative urgency (β = 0.08, 95% CI [0.00, 0.15], *p* = 0.04), and lack of premeditation (β = 0.08, 95% CI [0.01, 0.16], *p* = 0.03).

Finally, using the transdiagnostic dimensions extracted from factor analysis, we found that IIO was related to the date/delay effect (β = 0.11, 95% CI [0.03, 0.18], *p* = 0.008), while AD (β = 0.05, 95% CI [−0.03, 0.13], *p* = 0.21) and CIT (β = −0.01, 95% CI [−0.09, 0.06], *p* = 0.75) were not (Fig. 3d). This effect persisted when controlling for the other dimensions (Supplementary Table 9).

### Psychiatric symptom patterns and TD model fit

A lower model fit (i.e., higher WAIC) was significantly correlated with lack of perseverance (r = 0.08, *p* = 0.03; all other *p* < 0.05; Supplementary Table 10). In a single regression controlling for the influence of other scales, no relationship was significant (Supplementary Table 11). However, the relationship of model fit and lack of perseverance was also significant when controlling for gender, age and cognitive abilities (β = 0.08, 95% CI [0.00, 0.15], *p* = 0.04). All other relationships between model fit and symptom patterns/dimensions were insignificant (Supplementary Fig. 1a–b; Supplementary Tables 12–13).

### Psychiatric symptom patterns and the magnitude effect

Bayesian analysis of the magnitude effect revealed that the paired differences between log-transformed discount rates of small and medium (median paired difference = 0.62, 95% HDI [0.57, 0.66]) as well as medium and large (median paired difference = 0.37, 95% HDI [0.33, 0.41]) magnitudes had a probability of > 99.9% to be above zero. Moreover, the repeated-measures ANOVA was significant (*F*(1.86,1354.58) = 870.90, *p* < 0.001, η_p_² = 0.54; all pairwise comparisons *p* < 0.001). Thus, the magnitude effect was replicated, i.e., TD declined with larger reward magnitudes.

A more pronounced magnitude effect was correlated with higher scores in schizotypy (*r* = 0.08, 95% CI [0.01, 0.15], *p* = 0.03) (Fig. 2, Supplementary Table 14). Using a single regression, no predictor was significant (Supplementary Table 15). In regressions controlling for gender, age, and cognitive ability, we found depression/anxiety/stress (β = 0.09, 95% CI [0.01, 0.16], *p* = 0.03), schizotypy (β = 0.11, 95% CI [0.03, 0.19], *p* = 0.008) and ADHD (β = 0.08, 95% CI [0.00, 0.15], *p* = 0.048) to be significantly associated with the magnitude effect (Supplementary Fig. 1c, Supplementary Table 16).

Analyzing associations between the magnitude effect and transdiagnostic dimensions, we found that IIO (β = 0.09, 95% CI [0.01, 0.17], *p* = 0.03) was significantly related to the magnitude effect, while AD (β = 0.07, 95% CI [−0.01, 0.14], *p* = 0.07) and CIT (β = 0.06, 95% CI [−0.02, 0.14], *p* = 0.15) were not (Supplementary Fig. 1d). The effect did not persist when controlling for the other dimensions (Supplementary Table 17).

## Discussion

This study used a transdiagnostic approach to identify psychiatric symptom patterns associated with TD. Largely confirming our hypothesis, TD was higher in people scoring higher on depression/anxiety/stress, trait anxiety, OCD, schizotypy, ADHD, and different impulsivity constructs and lower in people with higher eating-related cognitive restraint. We did not find statistically significant associations for uncontrolled and emotional eating and autism. Analyses of transdiagnostic dimensions showed that higher TD was associated with dimensions representing AD and IIO. Moreover, we replicated the date/delay effect and found that people scoring higher in psychiatric symptom patterns loading on the IIO dimension (uncontrolled eating, emotional eating, ADHD, positive urgency, negative urgency, lack of premeditation) showed a stronger date/delay effect. Additionally, people scoring higher in lack of perseverance showed a worse TD model fit. Finally, the magnitude effect was replicated and people scoring higher in depression/anxiety/stress, schizotypy, and ADHD as well as IIO showed a stronger magnitude effect.

Our results are consistent with previous studies of aberrant TD in clinical populations with categorical diagnoses, replicating findings of both higher and shallower TD^7^ on a dimensional level. We thereby show that previous findings are not due to medication or treatment confounds. More generally, we provide evidence for the validity of a dimensional approach to mental disorders.

Moreover, the findings largely align with the only other transdiagnostic dimensional study examining TD^11^, confirming associations between TD and similar psychiatric symptom patterns (e.g., depression, anxiety) using different questionnaires. However, we significantly extend these findings by revealing associations with other psychiatric symptom patterns (e.g., schizotypy, impulsivity) and with transdiagnostic dimensions more closely resembling those found in previous transdiagnostic studies targeting different outcomes than TD^53–55,60,61^. Altogether, these results suggest that categorical findings are due to shared symptom dimensions, supporting the notion of TD as transdiagnostic marker. Specifically, the transdiagnostic nature of TD is reflected in anxiety/depression and inattention/impulsivity/overactivity but not in compulsion/intrusive symptoms. Future studies in clinical populations should explicitly test for associations between these symptom dimensions and TD^25^.

Potential mechanisms underlying these associations include impaired valuation and cognitive control systems, impaired future-oriented cognitive processing, ruminative cognition, or intolerance of uncertainty^7,11,62^. While we cannot provide a comprehensive explanation, associations of the IIO dimension with both the date/delay and magnitude effects highlight the potential importance of specific mechanisms and reveal potential intervention targets.

The association between TD and the IIO dimension may not be unexpected, since TD is commonly assessed as a measure of behavioral impulsivity and also referred to as choice impulsivity^63^. However, recent studies emphasized that the term *impulsivity* represents a highly heterogenous umbrella term, subsuming various phenomena^64–66^. This necessitates a clear distinction between different impulsivity-related constructs^66^. In the context of our study, it is therefore crucial to determine which mechanisms underlie the relatively small association between TD and self-reported IIO scores. While the mechanisms mentioned above may potentially all be relevant, our primary experimental manipulation highlights the importance of future-oriented cognitive processing. Specifically, the date manipulation, which has been associated with narrower time estimation and more episodic thinking^42,67^, was particularly effective in reducing TD in participants scoring high on the IIO dimension. Thus, it appears likely that higher TD in people who are more sensitive to the passage of time, e.g., impulsive individuals^68^, can be alleviated by experimentally enhancing episodic thinking, e.g., by using episodic tags^52,69^ or date cues^38–40^. Moreover, associations between the magnitude effect and IIO point towards another underlying mechanism. We speculate that this mechanism may be reflected in an aberrant self-control system because higher magnitude choices appear to rely on more self-control than lower magnitude choices^70^. Thus, an aberrant self-control system may be particularly detrimental in lower magnitude choices for people scoring higher on IIO, whereas higher magnitude choices may attenuate or alleviate these self-control impairments. Interestingly, this effect may also play a role in psychiatric dimensions underlying the AD factor (e.g., depression/anxiety/stress), although the association with AD itself did not reach significance. In sum, our findings suggest that both impairments in future-oriented cognitive processing and in self-control systems more generally may underlie the associations between TD and IIO. We would like to emphasize, however, that our interpretations are based on associations with two experimental effects only, one of which being the result of a non-preregistered explorative analysis, thus warranting replication. Furthermore, we only found evidence for mechanisms underlying the association between TD and IIO. That is, given that the date/delay effect and the magnitude effect did not significantly covary with AD, different mechanisms may (additionally) play a role. Future transdiagnostic studies could use additional experimental manipulations (e.g., of intolerance of uncertainty^71^) or examine neurobehavioral measures^62^ to identify the mechanisms underlying altered intertemporal decision-making in clinical and subclinical populations more precisely. If the mechanisms relevant to IIO discussed here are confirmed in future studies, this may suggest that interventions framing outcomes as psychologically near (date/delay effect) and large rewards (magnitude effects; also see^70^), may particularly alleviate aberrant TD in people scoring higher on IIO.

Several limitations should be considered. First, we did not collect information related to clinical diagnoses^12^. That is, as in previous studies with transdiagnostic approaches^53–55,60,61^, we recruited a large sample from the general population to examine psychopathology across a broad range of dimensional symptom patterns, but we did not screen participants for the presence or absence of psychiatric or neurological diagnoses, their developmental history or medication. Because of the lack of clinical information, inferences about clinical populations should be made with caution. Although previous studies found associations between temporal discounting and specific disorders^2–7^, the transdiagnostic significance of dimensions such as AD and IIO in clinical populations has yet to be confirmed. Specifically, it would be highly beneficial to investigate whether the transdiagnostic dimensions found to play a role in our study are confirmed in large clinical populations with different diagnostic categories (see, e.g.^72^, for an example of such a study in the context of goal-directed planning). Such studies may help to pinpoint the causes of clinically impaired decision-making and confirm the value of interventions such as the date/delay and magnitude effects whilst explicitly controlling for health-related third variables.

Second, given the correlation between AD and IIO, associations between TD and these dimensions are potentially confounded by the other dimension. Similar to comorbidities in categorical approaches, these dimensions are not entirely separable. Moreover, not directly health-related confounding third variables such as socio-economic status, other economic variables (such as risk perception) or, as outlined by Levitt and colleagues^11^, substance use (e.g., smoking status) could be relevant.

Third, our focus was entirely on monetary TD. However, as discussed recently^7^, other reward types may be more sensitive in specific populations. For example, associations between TD and emotional and uncontrolled eating may only be uncovered when using food instead of monetary rewards.

Overall, our study revealed positive associations of TD with psychiatric symptom patterns related to the dimensions AD and IIO and a negative association with eating-related cognitive constraints. These data support the view of TD as a transdiagnostic process across different mental disorders, although potential confounding variables should be considered. Importantly, the finding that people scoring high in IIO might be particularly influenced by the date manipulation and magnitude effects imply that clinical interventions targeting episodic processing and self-control systems could alleviate aberrant intertemporal decision-making in mental disorders characterized by inattention, impulsivity, and overactivity symptoms.

### Supplementary information

Supplementary Material

## Data Availability

Study materials as well as raw and processed datasets of the current study are available on the Open Science Framework (OSF) and accessible under the URL https://osf.io/ezaup/. The questionnaire data in this manuscript are also used for another transdiagnostic study relating complex decision-making based on the knapsack task to psychiatric symptom patterns. All main results reported here have not been submitted or published elsewhere.

## Code availability

The underlying code for the current study is available on the Open Science Framework (OSF) and accessible under the URL https://osf.io/ezaup/.
