# Supplementary material for: Association of temporal discounting with transdiagnostic symptom dimensions
Source: Npj Ment Health Res. 2024 Apr 16;3:13. doi: 10.1038/s44184-024-00060-3 (PMC11021403; doi:10.1038/s44184-024-00060-3)
Supplement: Supplementary file 1 — Supplementary Material [file 44184_2024_60_MOESM1_ESM.pdf]

Supplementary Material

Keidel K, Lu X, Suzuki S, Murawski C, & Ettinger U. Association of Temporal Discounting with Transdiagnostic Symptom Dimensions.

Content

Supplementary Methods **3**

Supplementary Tables **5**

Supplementary Table 1. Educational Level in the Adult Population in the United States (2021) and the Study Sample 5

Supplementary Table 2. Pearson Correlations between Log-Transformed Discount Rates ln(*k*) and Questionnaire Scores 6

Supplementary Table 3. Multiple Linear Regressions of Log-Transformed Discount Rates ln(*k*) on Questionnaire Scores (Including all Questionnaire Scores) 7

Supplementary Table 4. Multiple Linear Regressions of Log-Transformed Discount Rates ln(*k*) on Questionnaire Scores, Controlling for Gender, Age and Cognitive Abilities 8

Supplementary Table 5. Multiple Linear Regressions of Log-Transformed Discount Rates ln(*k*) on Transdiagnostic Factor Scores Controlling for Age, Gender, Cognitive Abilities and Factor Scores 9

Supplementary Table 6. Pearson Correlations between Date/Delay Effect and Questionnaire Scores 10

Supplementary Table 7. Multiple Linear Regression of Date/Delay Effect on Questionnaire Scores (Including all Questionnaire Scores) 10

Supplementary Table 8. Multiple Linear Regressions of Date/Delay Effect on Questionnaire Scores, Controlling for Gender, Age and Cognitive Abilities 11

Supplementary Table 9. Multiple Linear Regressions of Date/Delay Effect on Transdiagnostic Factor Scores Controlling for Age, Gender, Cognitive Abilities and Factor Scores 11

Supplementary Table 10. Pearson Correlations between Model Fit (WAIC) and Questionnaire Scores 12

Supplementary Table 11. Multiple Linear Regression of Model Fit (WAIC) on Questionnaire Scores (Including all Questionnaire Scores) 12

Supplementary Table 12. Multiple Linear Regressions of Model Fit (WAIC) on Questionnaire Scores and Transdiagnostic Dimensions, Controlling for Gender, Age and Cognitive Abilities 13

Supplementary Table 13. Multiple Linear Regressions of Model Fit (WAIC) on Transdiagnostic Factor Scores Controlling for Age, Gender, Cognitive Abilities and Factor Scores 13

Supplementary Table 14. Pearson Correlations between Magnitude Effect and Questionnaire Scores 14

Supplementary Table 15. Multiple Linear Regression of Magnitude Effect on Questionnaire Scores (Including all Questionnaire Scores)....14
Supplementary Table 16. Multiple Linear Regressions of Magnitude Effect on Questionnaire Scores, Controlling for Gender, Age and Cognitive Abilities....15
Supplementary Table 17. Multiple Linear Regressions of Magnitude Effect on Transdiagnostic Factor Scores Controlling for Age, Gender, Cognitive Abilities and Factor Scores....15
Supplementary Figures....16
Supplementary Fig. 1. Associations between Model Fit and Psychiatric Symptom Patterns and the Magnitude Effect and Psychiatric Symptom Patterns....16
Supplementary References....17

Supplementary Methods

Exclusion Criteria

As outlined in our preregistration, participants were excluded if they gave any nonsensical answers (see below for details). Pre-defined criteria beyond this (i.e., no reliable estimation of discount rates, answering “no” to control questions asking participants whether they will answer / answered honestly at the beginning or end of the study) did not apply and thus did not lead to any further exclusions.

Self-Report Psychometric Questionnaires

Participants completed the Depression Anxiety Stress Scales 21 (DASS-21)[1] assessing depression/anxiety/stress, the State-Trait Anxiety Inventory (STAI-T Y-2)[2] assessing trait anxiety, the Obsessive-Compulsive Inventory–Revised (OCI–R)[3] assessing obsessive-compulsive tendencies, the Short Oxford-Liverpool Inventory of Feelings and Experiences (sO-LIFE, without impulsive nonconformity)[4] assessing schizotypy, the Three-Factor Eating Questionnaire-R18 (TFEQ-R18)[5] assessing different types of disordered eating behavior (uncontrolled eating, cognitive restraint, emotional eating), the Autism-Spectrum Quotient Short (AQ-Short)[6] assessing autistic tendencies, the Adult ADHD Self-Report Scale V 1.1 (ASRS)[7] assessing ADHD symptoms, and the Short UPPS-P Impulsive Behavior Scale (sUPPS-P)[8] assessing impulsivity (negative urgency, lack of premeditation, lack of perseverance, sensation seeking, positive urgency).

Nonsensical Items

Participants were excluded if they gave an illogical response to at least one of five nonsensical items embedded within the questionnaires DASS-21[1], STAI-T Y-2[2], TFEQ-R18[5], AQ-Short[6], and the sUPPS-P[8]. Specifically, the nonsensical items were as follows (answer

options considered reasonable on a 4-point Likert scale in brackets): (1) “I have visited the planet Jupiter last year.” (1/2); (2) “I can understand at least one language in writing.” (3/4); (3) “I run two thousand miles every day to keep healthy.” (1/2); (4) “My body grows fifty centimeters per hour.” (1/2); (5) “I am awake at least one minute per week.“ (3/4).

Supplementary Tables

Supplementary Table 1. Educational Level in the Adult Population in the United States (2021) and the Study Sample

ISCED Category	ISCED Description	United States No. in 1,000 (%)	Study Participants No. (%)
0	(Less than) primary education	700 (0.3)	0 (0.0)
1	Primary education	7,531 (3.0)	12 (1.6)
2	Lower secondary education	16,092 (6.3)	3 (0.4)
3	Upper secondary education	74,530 (45.7)	149 (20.4)
4	Post-secondary non-tertiary education	42,099 (16.5)	109 (14.9)
5	Short-cycle tertiary education	25.388 (9.9)	31 (4.2)
6	Bachelor’s or equivalent level	56,350 (22.1)	310 (42.4)
7	Master’s or equivalent level	27,638 (10.8)	95 (13.0)
8	Doctoral or equivalent level	4,859 (1.9)	22 (3.0)

Note. Data from the United States are based on data from the U.S. Census Bureau[9] which we assigned to International Standard Classification of Education ISCED 2011 categories[10] based on the data mapping provided by the UNESCO (https://isced.uis.unesco.org/). Educational level in the study was assessed using the ISCED categories.

Supplementary Table 2. Pearson Correlations between Log-Transformed Discount Rates ln(*k*) and Questionnaire Scores

Variable	*r* (95% CI)	*p*	p_C
Correlations with ln(*k*) Delay			
Depression/Anxiety/Stress	.13 (.05 to .20)	< .001	< .001
Trait Anxiety	.12 (.05 to .19)	< .001	< .001
OCD	.13 (.06 to .20	< .001	< .001
Schizotypy	.11 (.04 to .18)	.004	.007
Uncontrolled Eating	.06 (-.01 to .14)	.08	.16
Cognitive Restraint	-.11 (-.18 to -.04)	.004	.007
Emotional Eating	.06 (-.02 to .13)	.13	.25
Autism	.01 (-.06 to .08)	.77	> .99
ADHD	.09 (.02 to .16)	.01	.02
Positive Urgency	.15 (.07 to .22)	< .001	< .001
Negative Urgency	.14 (.07 to .21)	< .001	< .001
Sensation Seeking	-.01 (-.08 to .07)	.84	> .99
Lack of Premeditation	.17 (.10 to .24)	< .001	< .001
Lack of Perseverance	.10 (.03 to .17)	.008	.02
Correlations with ln(*k*) Date			
Depression/Anxiety/Stress	.14 (.06 to .21)	< .001	< .001
Trait Anxiety	.14 (.07 to .21)	< .001	< .001
OCD	.11 (.03 to .18)	.004	.004
Schizotypy	.11 (.03 to .18)	.004	.007
Uncontrolled Eating	.03 (-.04 to .10)	.45	.45
Cognitive Restraint	-.08 (-.16 to -.01)	.02	.02
Emotional Eating	.05 (-.03 to .12)	.22	.25
Autism	.00 (-.07 to .07)	> .99	> .99
ADHD	.08 (.01 to .15)	.03	.03
Positive Urgency	.12 (.05 to .19)	.001	.001
Negative Urgency	.13 (.06 to .20)	< .001	< .001
Sensation Seeking	-.00 (-.08 to .07)	.94	> .99
Lack of Premeditation	.17 (.09 to .24)	< .001	< .001
Lack of Perseverance	.09 (.02 to .16)	.02	.02

Abbreviations: OCD, obsessive-compulsive disorder; ADHD, attention-deficit/hyperactivity disorder; CI, confidence interval; p_C, Bonferroni-Holm corrected *p*-value

Supplementary Table 3. Multiple Linear Regressions of Log-Transformed Discount Rates ln(*k*) on Questionnaire Scores (Including all Questionnaire Scores)

Variable	β (95% CI)	*p*	p_C	R^2	$F_{13,697}$	*p*
Regression of ln(*k*) Delay				.07	4.17	< .001
Depression/Anxiety/Stress	0.09 (-0.03 to 0.21)	.15	.15			
OCD	0.13 (0.02 to 0.24)	.02	.046			
Schizotypy	-0.00 (-0.14 to 0.13)	.97	> .99			
Uncontrolled Eating	0.01 (-0.11 to 0.13)	.90	.90			
Cognitive Restraint	-0.10 (-0.18 to -0.03)	.009	.02			
Emotional Eating	-0.02 (-0.13 to 0.08)	.68	> .99			
Autism	-0.10 (-0.20 to 0.00)	.06	.08			
ADHD	-0.09 (-0.21 to 0.03)	.16	.22			
Positive Urgency	0.08 (-0.02 to 0.19)	.11	.22			
Negative Urgency	0.04 (-0.06 to 0.15)	.43	.43			
Sensation Seeking	-0.04 (-0.11 to 0.04)	.37	.73			
Lack of Premeditation	0.12 (0.03 to 0.20)	.01	.01			
Lack of Perseverance	0.03 (-0.06 to 0.12)	.48	.95			
Regression of ln(*k*) Date				.06	3.84	< .001
Depression/Anxiety/Stress	0.13 (0.01 to 0.25)	.03	.07			
OCD	0.10 (-0.02 to 0.21)	.11	.11			
Schizotypy	0.02 (-0.11 to 0.16)	.76	> .99			
Uncontrolled Eating	-0.07 (-0.20 to 0.05)	.27	.53			
Cognitive Restraint	-0.07 (-0.14 to 0.01)	.08	.08			
Emotional Eating	0.02 (-0.09 to 0.13)	.73	> .99			
Autism	-0.11 (-0.21 to -0.01)	.04	.08			
ADHD	-0.10 (-0.23 to 0.02)	.11	.22			
Positive Urgency	0.03 (-0.07 to 0.13)	.53	.53			
Negative Urgency	0.08 (-0.03 to 0.18)	.16	.32			
Sensation Seeking	-0.03 (-0.11 to 0.05)	.49	.73			
Lack of Premeditation	0.14 (0.05 to 0.22)	.003	.006			
Lack of Perseverance	0.02 (-0.07 to 0.11)	.64	.95			

Abbreviations: OCD, obsessive-compulsive disorder; ADHD, attention-deficit/hyperactivity disorder; CI, confidence interval; p_C, Bonferroni-Holm corrected *p*-value

Supplementary Table 4. Multiple Linear Regressions of Log-Transformed Discount Rates ln(*k*) on Questionnaire Scores, Controlling for Gender, Age and Cognitive Abilities

**Variable**	**β (95% CI)**	***p***	***p*$_C$**
Regressions of ln(*k*) Delay			
Depression/Anxiety/Stress	0.11 (0.03 to 0.18)	.006	.006
Trait Anxiety	0.11 (0.04 to 0.19)	.004	.004
OCD	0.09 (0.01 to 0.17)	.02	.048
Schizotypy	0.09 (0.01 to 0.16)	.03	.052
Uncontrolled Eating	0.04 (-0.03 to 0.11)	.30	.60
Cognitive Restraint	-0.10 (-0.17 to -0.03)	.007	.01
Emotional Eating	0.05 (-0.02 to 0.12)	.14	.28
Autism	0.01 (-0.06 to 0.08)	.80	> .99
ADHD	0.09 (0.01 to 0.16)	.03	.06
Positive Urgency	0.11 (0.03 to 0.19)	.009	.02
Negative Urgency	0.10 (0.03 to 0.18)	.004	.008
Sensation Seeking	-0.02 (-0.10 to 0.05)	.53	> .99
Lack of Premeditation	0.15 (0.08 to 0.22)	< .001	< .001
Lack of Perseverance	0.08 (0.01 to 0.16)	.03	.068
Regressions of ln(*k*) Date			
Depression/Anxiety/Stress	0.12 (0.04 to 0.20)	.002	.004
Trait Anxiety	0.14 (0.06 to 0.22)	.001	.002
OCD	0.07 (-0.01 to 0.16)	.09	.09
Schizotypy	0.09 (0.01 to 0.16)	.03	.052
Uncontrolled Eating	0.01 (-0.07 to 0.08)	.84	.84
Cognitive Restraint	-0.08 (-0.15 to -0.01)	.04	.04
Emotional Eating	0.04 (-0.03 to 0.11)	.24	.28
Autism	-0.01 (-0.09 to 0.07)	.87	> .99
ADHD	0.07 (-0.01 to 0.15)	.09	.09
Positive Urgency	0.09 (0.01 to 0.17)	.03	.03
Negative Urgency	0.10 (0.03 to 0.18)	.006	.008
Sensation Seeking	-0.02 (-0.10 to 0.05)	.59	> .99
Lack of Premeditation	0.15 (0.08 to 0.22)	< .001	< .001
Lack of Perseverance	0.08 (0.00 to 0.15)	.051	.068

Note. Shown are only the coefficients for psychiatric symptom patterns. Each row represents a single regression, in which gender, age and cognitive abilities were controlled for. Abbreviations: OCD, obsessive-compulsive disorder; ADHD, attention-deficit/hyperactivity disorder; CI, confidence interval; *p*$_C$, Bonferroni-Holm corrected *p*-value

Supplementary Table 5. Multiple Linear Regressions of Log-Transformed Discount Rates ln(*k*) on Transdiagnostic Factor Scores Controlling for Age, Gender, Cognitive Abilities and Factor Scores

Variable	β (95% CI)	*p*	p_C	R^2	$F_{7,723}$	*p*
Regression of ln(*k*) Delay				.08	9.51	< .001
Gender1	0.01 (-0.13 to 0.15)	.91	> .99			
Gender2	-0.23 (-0.55 to 0.09)	.16	.33			
Age	-0.10 (-0.18 to -0.03)	.007	.01			
Cognitive Abilities	-0.24 (-0.31 to -0.16)	< .001	< .001			
AD	0.06 (-0.03 to 0.14)	.18	.18			
IIO	0.07 (-0.02 to 0.16)	.12	.23			
CIT	0.01 (-0.08 to 0.09)	.90	> .99			
Regression of ln(*k*) Date				.05	5.50	< .001
Gender1	0.05 (-0.10 to 0.19)	.51	> .99			
Gender2	0.02 (-0.35 to 0.40)	.91	.91			
Age	-0.07 (-0.15 to 0.01)	.07	.07			
Cognitive Abilities	-0.17 (-0.25 to -0.10)	< .001	< .001			
AD	0.09 (0.00 to 0.18)	.045	.09			
IIO	0.04 (-0.05 to 0.13)	.42	.42			
CIT	0.01 (-0.08 to 0.10)	.87	> .99			

Note. Gender1 and Gender2 represent two dummy variables referring to male and diverse, respectively (reference category: female). Abbreviations: AD, Anxious-Depression; IIO, Inattention, Impulsivity and Overactivity; CIT, Compulsive Behavior and Intrusive Thought; CI, confidence interval; p_C, Bonferroni-Holm corrected *p*-value

Supplementary Table 6. Pearson Correlations between Date/Delay Effect and Questionnaire Scores

Variable	*r* (95% CI)	*p*
Correlations with Date/Delay Effect		
Depression/Anxiety/Stress	.04 (-.03 to .12)	.23
Trait Anxiety	.04 (-.03 to .12)	.24
OCD	.05 (-.03 to .12)	.20
Schizotypy	.05 (-.02 to .13)	.16
Uncontrolled Eating	.12 (.05 to .20)	< .001
Cognitive Restraint	-.03 (-.11 to -.04)	.36
Emotional Eating	.09 (.02 to .16)	.02
Autism	.04 (-.04 to .11)	.29
ADHD	.07 (.00 to .15)	.046
Positive Urgency	.11 (.04 to .19)	.002
Negative Urgency	.09 (.01 to .16)	.02
Sensation Seeking	-.01 (-.08 to .06)	.81
Lack of Premeditation	.09 (.02 to .17)	.01
Lack of Perseverance	.03 (-.04 to .11)	.35

Abbreviations: OCD, obsessive-compulsive disorder; ADHD, attention-deficit/hyperactivity disorder; CI, confidence interval

Supplementary Table 7. Multiple Linear Regression of Date/Delay Effect on Questionnaire Scores (Including all Questionnaire Scores)

Variable	β (95% CI)	*p*	R^2	$F_{13,685}$	*p*
Regression of Date/Delay Effect			.03	1.46	.12
Depression/Anxiety/Stress	-0.01 (-0.14 to 0.12)	.87			
OCD	-0.00 (-0.11 to 0.11)	.97			
Schizotypy	-0.02 (-0.15 to 0.11)	.75			
Uncontrolled Eating	0.14 (0.01 to 0.26)	.03			
Cognitive Restraint	-0.05 (-0.13 to 0.03)	.18			
Emotional Eating	-0.04 (-0.15 to 0.08)	.51			
Autism	0.01 (-0.09 to 0.11)	.89			
ADHD	-0.00 (-0.13 to 0.12)	.98			
Positive Urgency	0.12 (0.00 to 0.23)	.048			
Negative Urgency	-0.04 (-0.17 to 0.09)	.54			
Sensation Seeking	-0.04 (-0.13 to 0.05)	.42			
Lack of Premeditation	0.04 (-0.05 to 0.13)	.43			
Lack of Perseverance	-0.01 (-0.10 to 0.08)	.84			

Abbreviations: OCD, obsessive-compulsive disorder; ADHD, attention-deficit/hyperactivity disorder; CI, confidence interval

Supplementary Table 8. Multiple Linear Regressions of Date/Delay Effect on Questionnaire Scores, Controlling for Gender, Age and Cognitive Abilities

Variable	β (95% CI)	*p*
Regressions of Date/Delay Effect		
Depression/Anxiety/Stress	0.04 (-0.04 to 0.12)	.34
Trait Anxiety	0.05 (-0.03 to 0.13)	.21
OCD	0.03 (-0.04 to 0.11)	.40
Schizotypy	0.05 (-0.02 to 0.13)	.17
Uncontrolled Eating	0.11 (0.04 to 0.19)	.003
Cognitive Restraint	-0.03 (-0.11 to 0.04)	.40
Emotional Eating	0.09 (0.01 to 0.16)	.02
Autism	0.06 (-0.01 to 0.13)	.10
ADHD	0.08 (0.00 to 0.16)	.046
Positive Urgency	0.10 (0.02 to 0.18)	.01
Negative Urgency	0.08 (0.00 to 0.15)	.04
Sensation Seeking	0.01 (-0.07 to 0.09)	.84
Lack of Premeditation	0.08 (0.01 to 0.16)	.03
Lack of Perseverance	0.03 (-0.04 to 0.11)	.42

Note. Shown are only the coefficients for psychiatric symptom patterns. Each row represents a single regression, in which gender, age and cognitive abilities were controlled for. Abbreviations: OCD, obsessive-compulsive disorder; ADHD, attention-deficit/hyperactivity disorder; CI, confidence interval

Supplementary Table 9. Multiple Linear Regressions of Date/Delay Effect on Transdiagnostic Factor Scores Controlling for Age, Gender, Cognitive Abilities and Factor Scores

Variable	β (95% CI)	*p*	R^2	$F_{7,710}$	*p*
Regression of Date/Delay Effect			.06	6.66	< .001
Gender1	-0.12 (-0.27 to 0.03)	.12			
Gender2	-0.61 (-0.94 to -0.28)	< .001			
Age	-0.04 (-0.12 to 0.04)	.37			
Cognitive Abilities	-0.19 (-0.26 to -0.12)	< .001			
AD	0.01 (-0.08 to 0.09)	.88			
IIO	0.12 (0.03 to 0.20)	.009			
CIT	-0.05 (-0.13 to 0.03)	.23			

Note. Gender1 and Gender2 represent two dummy variables referring to male and diverse, respectively (reference category: female). Abbreviations: AD, Anxious-Depression; IIO, Inattention, Impulsivity and Overactivity; CIT, Compulsive Behavior and Intrusive Thought; CI, confidence interval

Supplementary Table 10. Pearson Correlations between Model Fit (WAIC) and Questionnaire Scores

**Variable**	***r* (95% CI)**	***p***
Correlations with Model Fit (WAIC)		
Depression/Anxiety/Stress	-.01 (-.08 to .07)	.89
Trait Anxiety	-.05 (-.12 to .02)	.17
OCD	-.00 (-.08 to .07)	.96
Schizotypy	-.01 (-.08 to .07)	.88
Uncontrolled Eating	.03 (-.04 to .10)	.40
Cognitive Restraint	-.00 (-.07 to .07)	.97
Emotional Eating	.03 (-.05 to .10)	.49
Autism	-.00 (-.08 to .07)	.96
ADHD	-.01 (-.08 to .07)	.83
Positive Urgency	-.00 (-.07 to .07)	.97
Negative Urgency	.01 (-.07 to .08)	.89
Sensation Seeking	.02 (-.05 to .09)	.61
Lack of Premeditation	.05 (-.02 to .13)	.16
Lack of Perseverance	.08 (.01 to .15)	.03

Abbreviations: WAIC, widely applicable information criterion; OCD, obsessive-compulsive disorder; ADHD, attention-deficit/hyperactivity disorder; CI, confidence interval

Supplementary Table 11. Multiple Linear Regression of Model Fit (WAIC) on Questionnaire Scores (Including all Questionnaire Scores)

**Variable**	**β (95% CI)**	***p***	R^2	$F_{13,686}$	***p***
Regression of Model Fit (WAIC)			.01	0.62	.84
Depression/Anxiety/Stress	0.01 (-0.12 to 0.14)	.87			
OCD	0.01 (-0.10 to 0.12)	.90			
Schizotypy	-0.01 (-0.16 to 0.13)	.86			
Uncontrolled Eating	0.02 (-0.10 to 0.14)	.71			
Cognitive Restraint	0.00 (-0.08 to 0.08)	.93			
Emotional Eating	0.01 (-0.11 to 0.12)	.90			
Autism	0.01 (-0.09 to 0.11)	.89			
ADHD	-0.07 (-0.20 to 0.06)	.28			
Positive Urgency	-0.03 (-0.14 to 0.08)	.61			
Negative Urgency	0.00 (-0.13 to 0.13)	.98			
Sensation Seeking	0.03 (-0.06 to 0.11)	.53			
Lack of Premeditation	0.03 (-0.07 to 0.13)	.56			
Lack of Perseverance	0.09 (-0.00 to 0.19)	.053			

Abbreviations: WAIC, widely applicable information criterion; OCD, obsessive-compulsive disorder; ADHD, attention-deficit/hyperactivity disorder; CI, confidence interval

Supplementary Table 12. Multiple Linear Regressions of Model Fit (WAIC) on Questionnaire Scores and Transdiagnostic Dimensions, Controlling for Gender, Age and Cognitive Abilities

Variable	β (95% CI)	*p*
Regressions of Model Fit (WAIC)		
Depression/Anxiety/Stress	-0.02 (-0.10 to 0.06)	.65
Trait Anxiety	-0.06 (-0.14 to 0.02)	.14
OCD	-0.04 (-0.11 to 0.04)	.36
Schizotypy	-0.02 (-0.10 to 0.06)	.59
Uncontrolled Eating	0.01 (-0.06 to 0.09)	.74
Cognitive Restraint	0.00 (-0.07 to 0.08)	.90
Emotional Eating	0.03 (-0.05 to 0.10)	.45
Autism	0.00 (-0.07 to 0.08)	.91
ADHD	-0.01 (-0.09 to 0.07)	.80
Positive Urgency	-0.04 (-0.11 to 0.04)	.34
Negative Urgency	-0.02 (-0.10 to 0.05)	.54
Sensation Seeking	0.02 (-0.06 to 0.09)	.60
Lack of Premeditation	0.03 (-0.04 to 0.10)	.44
Lack of Perseverance	0.08 (0.00 to 0.15)	.04
AD	-0.04 (-0.11 to 0.04)	.36
IIO	0.02 (-0.06 to 0.09)	.69
CIT	-0.04 (-0.12 to 0.04)	.31

Note. Shown are only the coefficients for psychiatric symptom patterns. Each row represents a single regression, in which gender, age and cognitive abilities were controlled for. Abbreviations: WAIC, widely applicable information criterion; OCD, obsessive-compulsive disorder; ADHD, attention-deficit/hyperactivity disorder; AD, Anxious-Depression; IIO, Inattention, Impulsivity and Overactivity; CIT, Compulsive Behavior and Intrusive Thought; CI, confidence interval

Supplementary Table 13. Multiple Linear Regressions of Model Fit (WAIC) on Transdiagnostic Factor Scores Controlling for Age, Gender, Cognitive Abilities and Factor Scores

Variable	β (95% CI)	*p*	R^2	$F_{7,712}$	*p*
Regression of Model Fit (WAIC)			.07	8.07	< .001
Gender1	-0.02 (-0.17 to 0.13)	.78			
Gender2	0.08 (-0.40 to 0.55)	.75			
Age	-0.06 (-0.14 to 0.02)	.13			
Cognitive Abilities	-0.26 (-0.33 to -0.18)	< .001			
AD	-0.05 (-0.13 to 0.03)	.24			
IIO	0.05 (-0.03 to 0.13)	.23			
CIT	-0.04 (-0.13 to -0.04)	.28			

Note. Gender1 and Gender2 represent two dummy variables referring to male and diverse, respectively (reference category: female). Abbreviations: WAIC, widely applicable information criterion; AD, Anxious-Depression; IIO, Inattention, Impulsivity and Overactivity; CIT, Compulsive Behavior and Intrusive Thought; CI, confidence interval

Supplementary Table 14. Pearson Correlations between Magnitude Effect and Questionnaire Scores

**Variable**	***r* (95% CI)**	***p***
Correlations with Magnitude Effect		
Depression/Anxiety/Stress	.06 (-.01 to .14)	.09
Trait Anxiety	.05 (-.03 to .12)	.22
OCD	.04 (-.04 to .11)	.32
Schizotypy	.08 (.01 to .15)	.03
Uncontrolled Eating	.05 (-.02 to .13)	.15
Cognitive Restraint	.05 (-.02 to .13)	.15
Emotional Eating	.03 (-.04 to .11)	.36
Autism	.05 (-.03 to .12)	.22
ADHD	.07 (-.01 to .14)	.08
Positive Urgency	.01 (-.06 to .09)	.74
Negative Urgency	.03 (-.04 to .11)	.35
Sensation Seeking	.02 (-.05 to .10)	.51
Lack of Premeditation	-.01 (-.08 to .06)	.82
Lack of Perseverance	.01 (-.06 to .08)	.81

Abbreviations: OCD, obsessive-compulsive disorder; ADHD, attention-deficit/hyperactivity disorder; CI, confidence interval

Supplementary Table 15. Multiple Linear Regression of Magnitude Effect on Questionnaire Scores (Including all Questionnaire Scores)

**Variable**	**β (95% CI)**	***p***	R^2	$F_{13,692}$	***p***
Regression of Magnitude Effect			.01	0.68	.78
Depression/Anxiety/Stress	0.02 (-0.11 to 0.15)	.78			
OCD	-0.05 (-0.16 to 0.06)	.38			
Schizotypy	0.13 (-0.01 to 0.27)	.07			
Uncontrolled Eating	0.05 (-0.06 to 0.17)	.37			
Cognitive Restraint	0.04 (-0.04 to 0.12)	.29			
Emotional Eating	-0.04 (-0.16 to 0.07)	.47			
Autism	-0.00 (-0.10 to 0.10)	.97			
ADHD	-0.00 (-0.12 to 0.12)	.99			
Positive Urgency	-0.02 (-0.13 to 0.09)	.74			
Negative Urgency	-0.03 (-0.16 to 0.10)	.60			
Sensation Seeking	0.04 (-0.05 to 0.12)	.38			
Lack of Premeditation	-0.01 (-0.10 to 0.08)	.78			
Lack of Perseverance	-0.01 (-0.10 to 0.08)	.91			

Abbreviations: OCD, obsessive-compulsive disorder; ADHD, attention-deficit/hyperactivity disorder; CI, confidence interval

Supplementary Table 16. Multiple Linear Regressions of Magnitude Effect on Questionnaire Scores, Controlling for Gender, Age and Cognitive Abilities

Variable	β (95% CI)	*p*
Regressions of Magnitude Effect		
Depression/Anxiety/Stress	0.09 (0.01 to 0.16)	.03
Trait Anxiety	0.07 (-0.01 to 0.15)	.08
OCD	0.06 (-0.02 to 0.14)	.13
Schizotypy	0.11 (0.03 to 0.19)	.008
Uncontrolled Eating	0.06 (-0.01 to 0.14)	.11
Cognitive Restraint	0.05 (-0.03 to 0.12)	.20
Emotional Eating	0.04 (-0.04 to 0.11)	.33
Autism	0.05 (-0.02 to 0.13)	.13
ADHD	0.08 (0.00 to 0.15)	.048
Positive Urgency	0.03 (-0.04 to 0.11)	.36
Negative Urgency	0.06 (-0.02 to 0.13)	.13
Sensation Seeking	0.06 (-0.02 to 0.14)	.14
Lack of Premeditation	-0.00 (-0.08 to 0.07)	.89
Lack of Perseverance	0.03 (-0.05 to 0.10)	.47

Note. Shown are only the coefficients for psychiatric symptom patterns. Each row represents a single regression, in which gender, age and cognitive abilities were controlled for. Abbreviations: OCD, obsessive-compulsive disorder; ADHD, attention-deficit/hyperactivity disorder; CI, confidence interval

Supplementary Table 17. Multiple Linear Regressions of Magnitude Effect on Transdiagnostic Factor Scores Controlling for Age, Gender, Cognitive Abilities and Factor Scores

Variable	β (95% CI)	*p*	R^2	$F_{7,718}$	*p*
Regression of Magnitude Effect			.02	2.28	.03
Gender1	-0.08 (-0.23 to 0.07)	.28			
Gender2	0.09 (-0.39 to 0.57)	.72			
Age	0.12 (0.05 to 0.20)	.002			
Cognitive Abilities	0.03 (-0.05 to 0.10)	.51			
AD	0.03 (-0.06 to 0.11)	.53			
IIO	0.06 (-0.03 to 0.16)	.16			
CIT	0.03 (-0.05 to 0.12)	.45			

Note. Gender1 and Gender2 represent two dummy variables referring to male and diverse, respectively (reference category: female). Abbreviations: AD, Anxious-Depression; IIO, Inattention, Impulsivity and Overactivity; CIT, Compulsive Behavior and Intrusive Thought; CI, confidence interval

Supplementary Figures

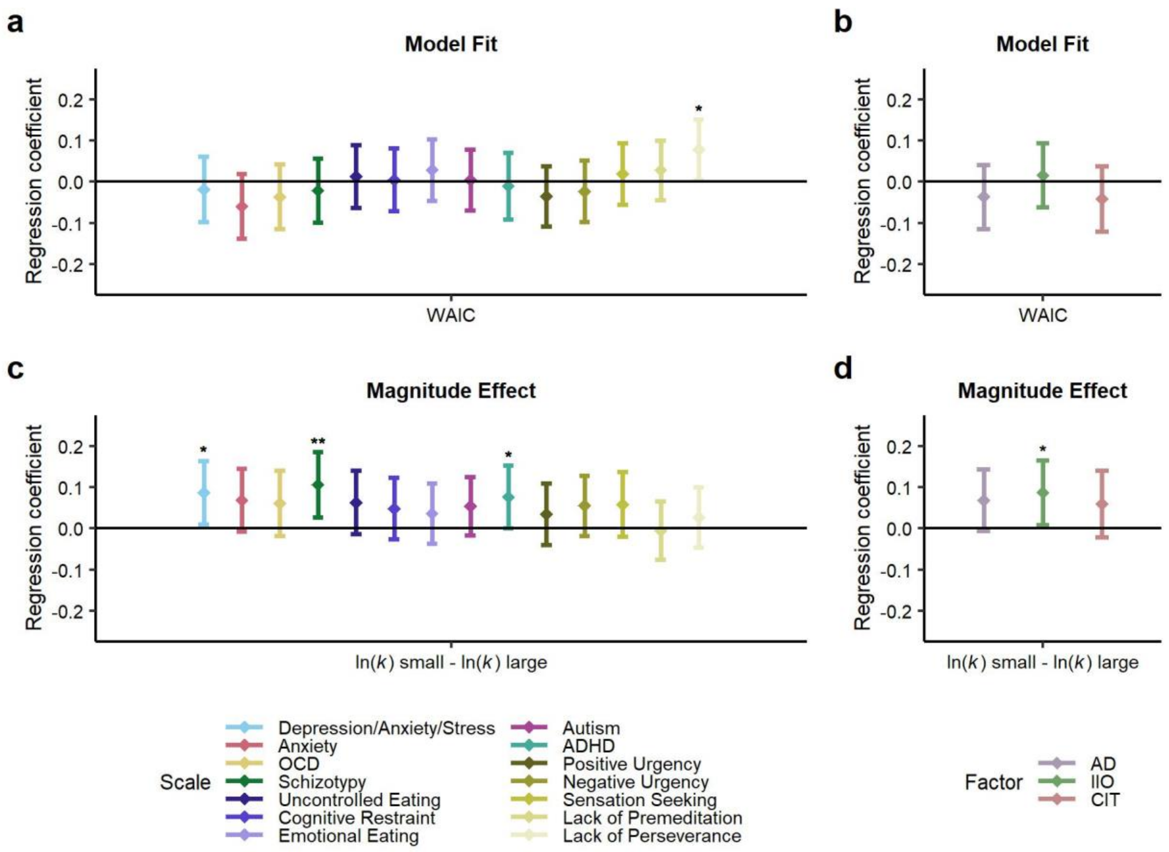

Supplementary Fig. 1. Associations between Model Fit and Psychiatric Symptom Patterns and the Magnitude Effect and Psychiatric Symptom Patterns. Presented are separate regressions of the temporal discounting model fit, i.e., WAIC (widely applicable information criterion), (a, b) and the magnitude effect (c, d) on different psychiatric symptom patterns (a, c) or transdiagnostic factors (b, d), controlling for gender, age and cognitive abilities. Error bars represent 95% confidence intervals. Abbreviations: OCD, obsessive-compulsive disorder; ADHD, attention-deficit/hyperactivity; AD, Anxious-Depression; IIO, Inattention, Impulsivity and Overactivity; CIT, Compulsive Behavior and Intrusive Thought

Supplementary References

1. Lovibond, P. F. & Lovibond, S. H. The structure of negative emotional states: Comparison of the Depression Anxiety Stress Scales (DASS) with the Beck Depression and Anxiety Inventories. *Behaviour Research and Therapy* **33**, 335–343 (1995).
2. Spielberger, C. D. *et al. Manual for the State-Trait Anxiety Inventory*. (Consulting Psychologists Press, 1983).
3. Foa, E. B. *et al.* The obsessive-compulsive inventory: Development and validation of a short version. *Psychol Assess* **14**, 485–496 (2002).
4. Mason, O., Linney, Y. & Claridge, G. Short scales for measuring schizotypy. *Schizophr Res* **78**, 293–296 (2005).
5. De Lauzon, B. *et al.* The Three-Factor Eating Questionnaire-R18 is able to distinguish among different eating patterns in a general population. *Journal of Nutrition* **134**, 2372–2380 (2004).
6. Hoekstra, R. A. *et al.* The construction and validation of an abridged version of the autism-spectrum quotient (AQ-short). *J Autism Dev Disord* **41**, 589–596 (2011).
7. Adler, L. A., Kessler, R. C. & Spencer, T. *Adult ADHD self-report scale-v1. 1 (ASRS-v1. 1) symptom checklist*. (World Health Organization, 2003).
8. Cyders, M. A., Littlefield, A. K., Coffey, S. & Karyadi, K. A. Examination of a short English version of the UPPS-P Impulsive Behavior Scale. *Addictive Behaviors* **39**, 1372–1376 (2014).
9. U.S. Census Bureau. *Table 1. Educational Attainment of the Population 18 Years and Over, by Age, Sex, Race, and Hispanic Origin: 2022*. https://www2.census.gov/programs-surveys/demo/tables/educational-attainment/2022/cps-detailed-tables/table-1-1.xlsx (2022).

10. United Nations Educational Scientific and Cultural Organization Institute for Statistics. *International Standard Classification of Education ISCED 2011*. (UNESCO, 2012).
